# Zinc and Calcium Cations Combination in the Production of Floating Alginate Beads as Prednisolone Delivery Systems

**DOI:** 10.3390/molecules25051140

**Published:** 2020-03-04

**Authors:** Paola Russo, Silvana Morello, Aldo Pinto, Pasquale Del Gaudio, Giulia Auriemma, Rita P. Aquino

**Affiliations:** Department of Pharmacy, University of Salerno, Via Giovanni Paolo II 132, I-84084 Fisciano (SA), Italy; paorusso@unisa.it (P.R.); smorello@unisa.it (S.M.); pintoal@unisa.it (A.P.); pdelgaudio@unisa.it (P.D.G.); aquinorp@unisa.it (R.P.A.)

**Keywords:** natural polysaccharides, alginate and divalent cations, laminar jet break-up, hollow and floating delivery systems, steroidal anti-inflammatory drugs

## Abstract

The aim of this research was to verify the application of alginate in combination with Ca^2+^ and Zn^2+^ ions to produce a floating and prolonged release system for the oral administration of prednisolone. Hollow and floating gel-beads were designed using prilling/ionotropic gelation as the microencapsulation technique, zinc acetate in the gelling solution as the alginate external crosslinker, and calcium carbonate in the feed as the internal crosslinking agent able to generate gas when in contact with the acidic zinc acetate solution. To achieve this goal, drug/alginate solutions were opportunely combined with different amounts of calcium carbonate. The effect of the addition of calcium carbonate into the feed solution on buoyancy, encapsulation efficiency, morphology, size distribution, as well as in vitro drug release profile of the alginate particles was studied. Moreover, the ability of the floating beads to modulate in vivo the anti-inflammatory response was assayed using the carrageenan-induced acute oedema in rat paw. The proposed strategy allowed obtaining alginate beads with extremely high encapsulation efficiency values (up to 94%) and a very porous inner matrix conferring buoyancy in vitro in simulated gastric fluid up to 5 h. Moreover, in vivo, the best formulation, F4, resulted in the ability to prolong the anti-inflammatory effect up to 15 h compared with raw prednisolone.

## 1. Introduction

In the field of oral controlled drug delivery, alginate is certainly one of the most explored natural polysaccharides owing to its cytocompatibility, biocompatibility, biodegradability, sol–gel transition properties, and chemical versatility that allows further changes to customize its drug release properties [1,2,3,4,5].

Alginate is a linear polysaccharide copolymer consisting of guluronic (G) and mannuronic acid (M) repeating units forming regions of M- and G-blocks and alternating structure (MG-blocks) [6]. Some divalent (Ca, Zn, and Ba) or trivalent (Fe(III) and Al) cations can interact with G-blocks of alginate in a highly cooperative manner, generating a 3D network according to the well-known ‘‘egg-box’’ model [7].

The prilling process (laminar jet break-up) has been widely used to produce crosslinked alginate micro-particles (gel-beads) acting as controlled drug delivery systems [8,9]. Prilling is a mild and scalable microencapsulation technique working essentially through two steps: the breaking apart of a laminar jet of alginate solution into a row of mono-sized drops through a vibrating nozzle and the successive drops’ ionotropic gelling in a solution of cations acting as crosslinkers. In the last few years, a large variety of gel-beads has been produced, varying the types of polysaccharides (mainly alginate, pectin, chitosan, and so on) used alone or in combination [10,11]; the configuration of the prilling apparatus to obtain ‘only core’ or core-shell beads [12]; and the gelling conditions in terms of cross-linker, pH of the reticulation solution, and gelling time [13,14].

Moreover, calcium alginate has often been used in combination with different gas-generating agents such as sodium bicarbonate, calcium carbonate, and citric acid or tartaric acid to obtain low-density floating formulations [15,16,17]. Systems floating in gastric medium can remain in the stomach for a prolonged period after oral administration; release the drug slowly [18,19,20]; and extend its in vivo effect, increasing both its therapeutic efficacy and safety with substantial benefits and a better patient compliance [21]. In particular, this approach can be interesting for API (active pharmaceutical ingredient) with a short half-life such as prednisolone (SAID, steroidal anti-inflammatory drug), useful to treat some chronic inflammatory diseases, which gain benefits from the reduction of the daily dose administered and correlated reduction of the side effects [13].

Although some studies have been focused on the production of floating alginate beads with calcium in the gelling solution, few data are available on the simultaneous use of two different cations as cross-linking agents [22,23]. Moreover, no results have been reported on the exploitation of two different cations in a double simultaneous gelation mechanism, that is, external, promoted by zinc ions in the gelling solution, and internal, promoted by calcium ions in prilling the feed solution.

As previously reported in our study [22], in fact, the co-presence of calcium (Ca^2+^) and zinc (Zn^2+^) could have a synergistic effect during the external ionotropic gelation, significantly improving the technological properties (i.e., encapsulation efficiency and drug release control) of the resulting cross-linked alginate beads. This effect is because of both the ability of Ca^2+^ to establish quicker electrostatic interactions with guluronic groups and the ability of Zn^2+^ to bond in covalent-like manner carboxylate groups of guluronic and mannuronic moieties of alginate.

In this paper, a double internal–external alginate gelation approach was investigated, verifying the combined effect of Ca^2+^ in the feed and Zn^2+^ in the gelling medium on the final beads’ performance. With this in mind, floating Ca-Zn alginate beads were designed as potential oral prolonged delivery systems of prednisolone as a model SAID, taking advantage of the efficient prilling/ionotropic gelation in the presence of zinc ions [22], widely known as the promoter of the external alginate gelation, and the pore-generating ability of calcium carbonate. Among different gas-generating agents employed for the formulation of floating beads [24,25], calcium carbonate was selected for its capability to interact with alginate guluronic groups, potentially responsible for an additional internal gelation when added in the feed.

Drug/alginate solutions (alginate 2.5% *w*/*v* and drug/polymer ratio 1:5) [22] were opportunely combined with calcium carbonate and processed by prilling to obtain hollow gel beads, after drip into the zinc acetate solution. CO_2_ exploited during the particle manufacturing gave a porous alginate matrix with good results in terms of the encapsulation efficiency and morphology of the beads. The beads were shown to float in simulated gastric medium and to modulate in vivo the anti-inflammatory response of prednisolone, tested in a rat paw oedema model.

## 2. Results and Discussion

### 2.1. Floating Beads’ Production and Characterization

Six floating Ca-Zn alginate formulations loaded with prednisolone were produced, adding CaCO_3_ in the polymer/drug feed solution as the gas-generating agent. When feed dripped in zinc acetate medium (pH 1.5), carbonate produced CO_2_ that was entrapped in the forming hydrogel matrix, reducing the system density [24,26,27]. The effect of the addition of different amounts of calcium carbonate into the feed solution on particle encapsulation efficiency, morphology, size distribution, as well as in vitro drug release profile was studied.

The results reported in Table 1 show that all polysaccharide-based beads produced in the selected and optimized prilling operative conditions were in a narrow size distribution, with mean diameter values ranging from 1.6 mm (#F4) to 2.5 mm (#F1). The first interesting result regards the very high encapsulation efficiency obtained for porous beads F2–F6 (87.8–94.5%), even higher than the non-porous beads F1 (78.6%), obtained without calcium carbonate. This unexpected effect could be explained by the change of calcium carbonate solubility when it moves from the alginate solution to the acidic gelling bath. This salt is very low soluble at a neutral pH (water solubility 15 mg/L); during the ionotropic gelation process, the alginate-CaCO_3_/drug droplets produced by prilling apparatus fall down into the zinc acetate solution. Here, the released Ca^2+^ ions can promote an internal gelation, by cross-linking the alginate carboxyl group [24,27], which, in addition to the external gelation obtained by Zn^2+^ ions, prevented the leakage of the drug during the gel particle formation, with an increase of encapsulation efficiency (EE).

**Figure 1:**
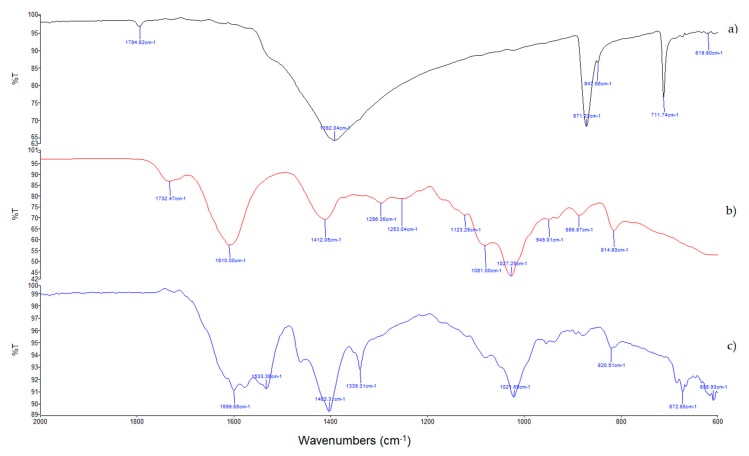
Fourier transform infrared spectroscopy (FTIR) spectra of CaCO_3_ (**a**), non-floating blank F1 beads (F1_b) (**b**), and floating blank F4 beads (F4_b) (**c**).

The presence of calcium in the matrix of F4 beads was confirmed by IR analyses (Figure 1) on calcium carbonate, F1_b beads (produced without calcium carbonate), and #F4_b (produced from a solution containing alginate and calcium carbonate in a 1:0.50 *w*/*w* ratio).

In #F1_b and #F4_b spectra, asymmetric (1610 and 1600 cm^−1^) and symmetric (1412 and 1403 cm^−1^) stretching vibrations of carboxylate ion were detected [28]; the pick broadening observed in #F4_b compared with #F1_b is because of the interaction of carboxylate ions with both zinc and calcium cations with different charge density, radius, and atomic weight [29]. In addition, the #F4_b spectrum evidenced new peaks at a lower wave number observed in calcium acetate spectra [30] attributed to the asymmetric C-O stretching vibrations (1533 cm^−1^, ν_as_(C-O)) cm^−1^) and symmetric methyl bending vibration (1339 cm^−1^, δ_s_ (CH_3_)).

In order to evaluate the effect of calcium carbonate on the inner structure of the bead formulations, they were cryo-fractured and analysed by scanning electron microscopy (SEM).

**Figure 2:**
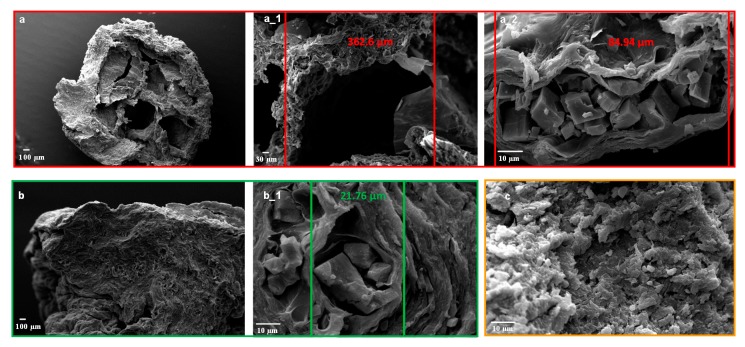
Scanning electron microscopy (SEM) microphotographs of cryo-fractured zinc-alginate beads: F6 (**a**,**a_1**,**a_2**), F4 (**b**,**b_1**), and F1 (**c**) at different magnifications.

Figure 2 shows the differences between the inner matrix of #F6 and #F4, obtained with a 1:1 and 1:0.5 alginate/calcium carbonate ratio, respectively; #F6 loading a greater amount of CaCO_3_ displayed the formation of a macro-porous structure (#F6, Figure 2a,a_1) owing to a higher quantity of CO_2_ produced during the gelling phase.

At the same time, it is possible to detect the presence of a micro-porous structure for both formulations, whose porous diameters decreased significantly from #F6 to #F4 (84.94–21.76 µm, respectively, Figure 2a_2,b_1). F4 resulted in a more compact inner structure without empty macro-porous (Figure 2b), as confirmed by the lower particle diameter measured.

To verify whether the high porous nature of the matrix led particles to float over the gastric fluids, the buoyancy test in simulated gastric fluid (SGF) was performed.

**Figure 3:**
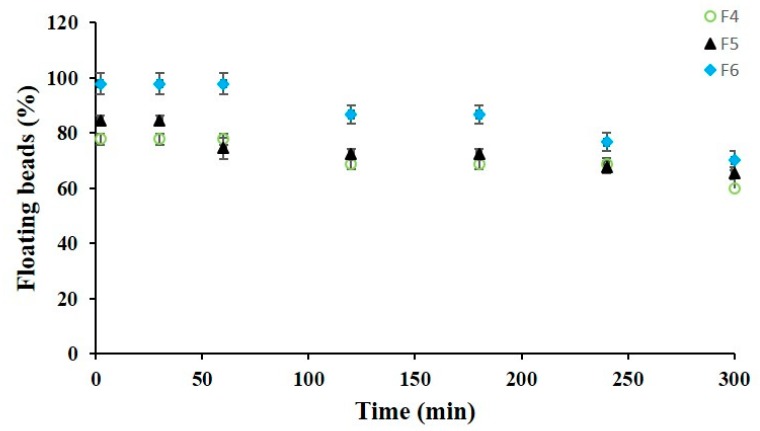
Floating percentages of #F4, #F5, and #F6 in simulated gastric fluid (SGF).

Interestingly, #F4, #F5, and #F6 were able to float in SGF up to 5 h after an initial floating lag time of 2 min and without any particles disintegration or erosion. Buoyancy ability was directly related to the concentration of the gas-generating agent into the feed solutions; increasing CaCO_3_ from 1.25% to 2.5% *w*/*v* (Alg/CaCO_3_ mass ratio from 1:0.5 to 1:1; Table 1), beads’ porosity as well as floating properties increased. In fact, the percentage of particles able to float in acidic medium (t = 2 min) was 77.8% ± 1.9% for #F4, 84.4% ± 1.9% for #F5, and reaching 97.8% ± 3.8% for formulation F6. These percentages of floating beads continued to be high up to 300 min (Figure 3), set as the floating time (FT), as after this, more than 50% of the particles sink.

As a confirmation, #F2 and #F3 (Alg/CaCO_3_ mass ratio of 1:0.1 and 1:0.25, respectively) resulted the inability to float in SGF, owing to a low calcium carbonate concentration giving a low porosity of the matrix.

### 2.2. In Vitro Prednisolone Release from Dried Beads

In order to evaluate the potential ability of the floating particles to control Pred release, dissolution experiments were performed in SGF for 5 h, time-lapse correspondent to the maximum of the floating time.

**Figure 4:**
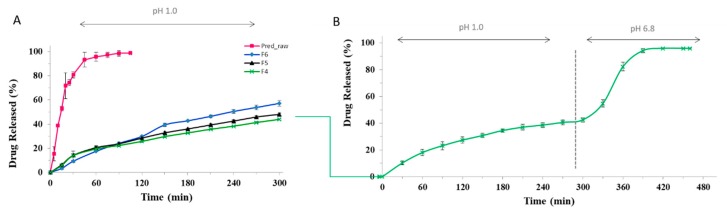
(**A**) Release profiles of Pred raw material compared with formulations F4, F5, and F6 in SGF for 5 h; (**B**) release profile of #F4 obtained with a change pH assay.

The three floating formulations, F4, F5, and F6, showed a slow and prolonged prednisolone release in SGF with respect to raw Pred. Particularly, #F4 exhibited a cumulative drug release of 22% at t = 120 min and 43% at t = 300 min, compared with #F5 and #F6 at the same times (Figure 4, Panel A). All F4, F5, and F6 formulations were able to control the drug release in acidic medium for the entire time corresponding to the floating period (5 h), suggesting that the tougher matrix obtained thanks to the double gelation process (internal and external) promoted by Ca and Zn ions, respectively, although porous, is able to reduce swelling and erosion processes in SGF.

For non-floating and non-porous alginate beads F1 (produced without calcium carbonate), a diffusional mechanism of Pred controlled by hydration, swelling, and then erosion phenomena, typically occurring in a cross-linked alginate matrix, has been previously demonstrated [31]. The drug release kinetic of the formulation F4 with the best ability to control Pred release in SGF for 5 h and a good in vitro buoyancy (FP of 77.8%) was further investigated in SIF, where it reached the 100% of Pred released 90 min after the pH change (Figure 4, Panel B). A similar trend, attributed to a rapid matrix erosion in pH 6.8 phosphate buffer, was observed for #F5 and #F6 (data not shown).

The formulation F4 was selected for the in vivo experiments, to verify whether its in vitro properties can induce a prolonged anti-inflammatory effect in rats.

### 2.3. In Vivo Experiments

The in vivo anti-inflammatory effect of #F4, in comparison with raw prednisolone, was evaluated using a modified protocol of carrageenan-induced oedema in rat paw (male Wistar rats). Pred was administered at 0.5, 5, or 15 h before oedema induction and at the dose of 3.0 mg/kg. The dose of 3.0 mg/kg of Pred was selected based on dose-response experiments conducted in our previous research [31].

**Figure 5:**
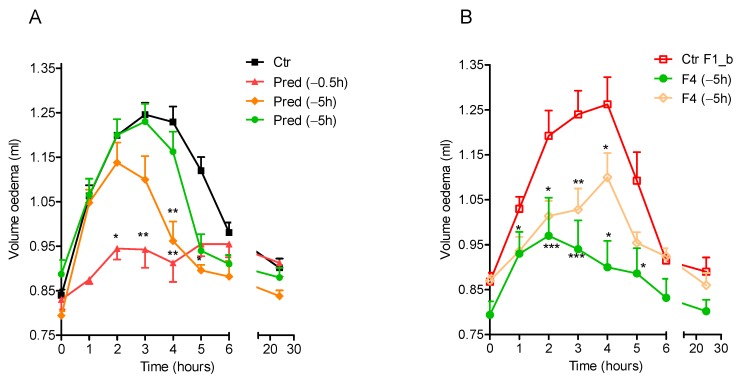
Oedema volume reduction obtained by administering per os pure prednisolone, Pred (Panel A), and #F4 (Panel B) at different time points (0.5, 5, or 15 h) to rats before carrageenan injection, compared with the control; mean ± SEM (*n* = 4 animals per group). Panel A: black square → control group, Ctr, receiving as control 1 mL of methylcellulose (MC) 0.5% w/v; red triangle → group Pred (−0.5 h), receiving prednisolone half an hour before carrageenan injection; orange diamond → group Pred (−5 h), receiving prednisolone 5 h before carrageenan injection; green circle → group Pred (−15 h) receiving prednisolone 15 h before carrageenan injection. Panel B: empty red square → control group, Ctr F1_b, receiving as control blank beads; green circle → group F4 (−5 h), receiving #F4 5 h before carrageenan injection; empty pink diamond → group F4 (−15 h), receiving #F4 15 h before carrageenan injection. *p < 0.05, **p < 0.01 compared to control.

The inflammation induced by carrageenan injection caused an increase in the rat paw volume (oedema) compared with the baseline one. According to literature data [32], the maximum volume of the oedema was observed in the control group (Ctr Panel A, Figure 5) 3 h after the injection of the phlogistic agent.

As shown in Figure 5, Panel A, raw prednisolone was able to suppress the inflammation and to significantly reduce oedema volume only when administered 0.5 h before carrageenan injection, but failed to control the oedema when administered to rats 5 h or 15 h before carrageenan injection. However, when this API was orally administered to rats through the formulation F4, acting in vitro as a prolonged release system, an extended anti-inflammatory activity was observed. In fact, as shown in Figure 5 Panel B, #F4 was still able to control oedema even when administered to rats 15 h before the phlogistic agent injection (control group: blank beads).

Indeed, F4 beads with a very tough Ca-Zn-alginate matrix are an interesting delivery system, showing both the right porosity, required for buoyancy, and the ability to control the drug release in SGF, with an impressive extension of the in vivo anti-inflammatory activity.

## 3. Materials and Methods

### 3.1. Materials

Sodium alginate (ALG_European Pharmacopoeia X, viscosity 250–300 cP, 1% in H_2_O at 20 °C, MW ~240 KDa) was supplied from Carlo Erba (Carlo Erba, Milan, Italy).

Calcium carbonate, zinc acetate dehydrate, and prednisolone (Pred) were purchased from Sigma-Aldrich (Sigma-Aldrich, Milan, Italy). All other chemicals and reagents were obtained from Sigma Aldrich and used as supplied.

### 3.2. Methods

#### 3.2.1. Beads’ Production

For the production of F1–F6 beads (Table 1), the alginate concentration (2.5% *w*/*v*) and drug/polymer ratio (Pred/alginate ratio 1:5) in the feed solution were fixed on the basis of our previous research, as well as zinc acetate dihydrate content (0.5 M) in the gelling solution [31]. In brief, sodium alginate was dissolved in distilled water at room temperature under gentle stirring for 18 h in order to obtain 100 mL of the polymer solution. Prednisolone and different amount of calcium carbonate (alginate/calcium carbonate ratios from 0.1:1 to 1:1) were successively suspended into the polymer solution and stirred for 2 h.

Beads were manufactured by a vibrating nozzle device (Nisco Encapsulator Var D; Nisco Engineering Inc., Zurich, Switzerland), equipped with a syringe pump (Model 200 Series, Kd Scientific Inc., Boston, MA, USA), pumping the drug/polymer/carbonate liquid feed through a 600 µm nozzle. The experiments were performed at a flow rate of 6 mL/min. The vibration frequency used to break up the laminar liquid jet was set at 350 Hz, at an amplitude of vibration of 100%. The distance between the vibrating nozzle and the gelling bath was fixed at 25 cm. The generated drops were observed as a stationary chain of drops by means of an LED-stroboscopic light, automatically synchronized from the control cabinet with the adjusted vibration frequency. Drug/polymer/carbonate droplets fell into the Zn^+2^ aqueous solutions (pH adjusted to 1.5 adding an adequate amount of a 1 N solution of HCl), where they immediately gelified under gentle stirring.

In detail, the syringe pump was stopped after processing 10 mL aliquots of the feed solution and the formed beads were kept in the gelling solution for a medium time of 2 min. Then, the crosslinked particles were recovered and thoroughly rinsed with distilled water. Thereafter, the syringe pump was re-started and the generated droplets were collected in a new gelling bath. Experiments were performed, processing few mL of feed solution to guarantee a gelling time as similar as possible for all particles. This procedure was repeated several times in order to obtain the desired amount of particles.

After production, the gel beads were left to air dry at room conditions, until a constant weight was reached (about 12 h). As a comparison, unloaded beads (blank) were produced following the same protocol.

#### 3.2.2. Drug Content and Encapsulation Efficiency

Accurately weighed amounts of beads from each manufactured batch (about 10 mg each) were dissolved under vigorous stirring in PBS buffer (100 mM, pH 7.0) in order to disintegrate the polymer matrix and release the encapsulated drug. Afterwards, 23 mL of ethanol was added and the suspension was centrifuged at 6000 rpm for 15 min.

The actual drug content (ADC) was determined by UV spectroscopy (Evolution 201 UV/VIS Spectrometer; Thermo Scientific, Waltham, MA) at λ of 244 for prednisolone quantification using the following equation:

(1) $$ADC\left(\%\right)=\frac{drug\;content\;in\;dry\;beads}{weight\;of\;dry\;beads\;}\times 100$$

Encapsulation efficiency (EE) was calculated as the ratio actual to theoretical drug content (TDC), that is, the weight of drug added (g)/weight of polymers/excipients and drug added (g) × 100. Each analysis was performed in triplicate; the results were expressed in terms of mean ± standard deviation.

#### 3.2.3. Beads’ Size, Size Distribution, and Morphology

The dimensional distribution and morphology of dried beads were studied by scanning electron microscopy (SEM) using a Carl Zeiss EVO MA 10 microscope with a secondary electron detector (Carl Zeiss SMT Ltd., Cambridge, UK) equipped with a LEICA EMSCD005 metallizator, producing a deposition of a 200–400 Å thick gold layer. The analysis was conducted at 20 KeV. The projection diameter of dried beads was obtained by image analysis (Image J software, Wayne Rasband, National Institute of Health, Bethesda, MD, USA). About one-hundred-bead micrographs were analysed for each preparation and for at least three different prilling processes. Inner structure images were obtained by cryofracture of dried beads and further analysis by SEM, as previously reported [13]. In brief, freeze-fracture of the beads was performed by plunging the particles into liquid nitrogen for 1 min, and then fracturing the frozen beads by means of two needles. Split particles were attached to the aluminum stab, covered with gold, and analyzed.

#### 3.2.4. Fourier Transform Infrared Spectroscopy (FTIR) Analyses

FTIR spectra between 2000 and 600 cm^−1^ were recorded using an FTIR spectrophotometer (IRAffinity-1S, Shimadzu Corporation, Kyoto, Japan) equipped with a MIRacle ATR accessory with a ZnSe crystal plate. The samples were directly analyzed using 256 scans with a 1 cm^−1^ resolution step.

#### 3.2.5. In Vitro Buoyancy Test

Floating properties of prednisolone hollow beads were evaluated through the USP dissolution Apparatus II (Sotax AT7 Smart—Sotax, Allschwil, Switzerland). About 100 mg (≈ 30 beads per formulation) were placed into 1 L glass vessels filled with 750 mL of simulated gastric fluid (SGF 0.1 M hydrochloric acid; pH 1; 75 rpm −37 °C) and the time the beads took to surface and float on the SGF medium was considered as the floating lag time (FLT). Moreover, throughout the buoyancy test, the floating percentage (FP) was calculated as the ratio between the number of floating beads and the total number of beads inserted in each vessel [33].

#### 3.2.6. Drug Release Studies

In vitro drug release tests were conducted in sink conditions on given amounts of bead formulations using a USP 27 dissolution apparatus II: paddle, 100 rpm, 37 °C (Sotax AT7 Smart—Sotax, Allschwil, Switzerland) on line with a UV spectrophotometer (Lambda 25 UV/VIS Spectrometer, Perkin Elmer, Waltham, MA, USA) with an automated cuvette charger.

Briefly, floating beads were added to the simulated gastric fluid (SGF, 0.1 M HCl) for 5 h, the floating time evidenced for #F4–#F6 formulations. The release kinetic of the formulation F4, selected for the in vivo study, was further investigated via a pH-change assay: particles were initially plunged into 750 mL of a 0.1 M solution of HCl; after 5 h, 250 mL of 0.2 M Na_3_PO_4_ was added into the acidic medium and the pH was adjusted to 6.8, continuously monitoring Pred release.

Data were analyzed spectrophotometrically at a λ of 244 nm for Pred quantification. Dissolution tests were conducted on six different batches of particles; mean values and standard deviation were reported.

#### 3.2.7. Carrageenan Oedema Induction

Male Wistar rats (180–220 g) were purchased from Charles River (Charles River Laboratories, Calco, Italy). To carry out the carrageenan oedema induction, rats were anaesthetized with isoflurane and oedema was induced by injecting 100 µL of carrageenan 1% (*w*/*v*) in the right hind paw, as previously reported [14]. The intensity of the oedema as well as its duration was assessed by rat paw volume measurements using plethysmometer (2Biological-Instruments, Italy) at time zero every hour up to 6 h and at 24 h after carrageenan injection. Animals were then sacrificed. Data were expressed as mean ± S.E.M. Statistical differences were evaluated by two-way analysis of variance (ANOVA) followed by Bonferroni posttest. *p*-value < 0.05 was considered statistically significant. All the experiments were approved by Italian Health Ministry (authorization n° 805/2015-PR) and conducted according to institutional animal care guidelines, Italian Law 26/2014 based on the European Community Law for Animal Care 2010/63/UE.

#### 3.2.8. In vivo Experiments

To assess the prolonged in vivo anti-inflammatory efficacy of #F4 in comparison with raw prednisolone, rats were treated by oral gavage with #F4 (Pred equivalent dose 3 mg kg^−1^) or with raw prednisolone [34], using the same doses. Samples were administered to rats in 1 mL of methylcellulose (MC) 0.5% *w*/*v* at the time 0.5, 5, or 15 h before the injection of the phlogistic agent. Control groups received 1 mL of MC or blank beads in 1 mL of MC by oral gavage. In detail, rats were randomly divided into seven groups: (1) Control group (Ctr), receiving 1 mL of MC 0.5% *w*/*v*; (2) Group P (−0.5 h), receiving Pred half an hour before carrageenan injection; (3) Group P (−5 h), receiving Pred 5 h before carrageenan injection; (4) Group P (−15 h), receiving Pred 15 h before carrageenan injection; (5) Group (Ctr F1_b), receiving blank beads; (6) Group F4 (−5 h), receiving #F4 5 h before carrageenan injection; (7) Group F4 (−15 h), receiving #F4 15 h before carrageenan injection.

Data were analysed by two-way ANOVA followed by Bonferroni post-test to compare each group to its control.

## 4. Conclusions

Hollow alginate particles able to float and at the same time to prolong prednisolone release in simulated gastrointestinal fluids were obtained by prilling, starting from an optimized Zn-alginate formulation (alginate 2.5% *w*/*v*, drug/Pred ratio 1:5) with the addition of calcium carbonate, as the gas-generating agent and internal gelation promoter during the beads’ production. The opportune amount of the gas-generating and cross-linking agents allowed obtaining porous polysaccharide-based beads able to encapsulate a high amount of SAID (EE up to 94%) and to control prednisolone delivery efficiently. The prolonged anti-inflammatory effect observed up to 15 h in rats, compared with prednisolone raw, is a proof of the in vivo efficiency of the system.

This alginate-based system could be proposed as an interesting technological platform able to extend the anti-inflammatory efficacy of steroidal anti-inflammatory drugs such as prednisolone (characterized by high efficacy and high tolerability, but short half-life) for many hours and successfully treat patient suffering from chronic inflammatory diseases, reducing the frequency of the oral administration.

## Figures and Tables

**Table 1:** Alginate/calcium carbonate floating beads loaded with prednisolone. Formulation code, alginate concentration, alginate/calcium carbonate ratio, encapsulation efficiency (EE), and mean diameter.

Formulation Code	Alginate Concentration (*w*/*v*)	Alg/CaCO_3_ Ratio (*w*/*w*)	EE (% ± SD)	Mean Diameter (mm ± SD)
**F1**	2.50%	/	78.6 ± 2.1	2.50 ± 0.12
**F2**		1:0.10	88.4 ± 2.9	2.20 ± 0.09
**F3**		1:0.25	94.5 ± 1.7	2.23 ± 0.11
**F4**		1:0.50	87.8 ± 3.5	1.63 ± 0.07
**F5**		1:0.75	91.1 ± 2.1	1.94 ± 0.14
**F6**		1:1.00	91.1 ± 2.0	2.28 ± 0.15
